# The relationship between social support and goal pursuit among Chinese college students: The mediating role of just-world beliefs

**DOI:** 10.3389/fpsyg.2022.1051884

**Published:** 2022-12-15

**Authors:** Dan Chen, Xiaolong Miao, Yuning Ma, Yulong Tang

**Affiliations:** ^1^Research Center for Socialism With Chinese Characteristics, Zhejiang University, Hangzhou, China; ^2^School of Marxism, Zhejiang University, Hangzhou, China; ^3^Institute of Applied Psychology, College of Education, Zhejiang University of Technology, Hangzhou, Zhejiang, China; ^4^Department of Psychology and Behavioral Sciences, Graduate School, Zhejiang University, Hangzhou, Zhejiang, China

**Keywords:** social support, just-world beliefs, goal pursuit, college students, China

## Abstract

Based on the self-determination theory, this study examined the mechanisms underlying the influence of social support on goal pursuit among college students and focused on the mediating role played by just-world beliefs. The Aspiration Index Scale, Just-World Beliefs Scale, and Perceived Social Support Scale were used to assess relationships among 424 college students’ just-world beliefs, social support, and goal pursuit; additionally, the underlying mechanisms of those relationships were examined. The results showed that (1) there was a significant positive correlation between social support, just-world beliefs, and goal pursuit; (2) social support had a significant positive influence on goal pursuit; (3) just-world beliefs played a fully mediating role in the influence of social support on goal pursuit, with a mediating effect of 36.38%; and (4) personal just-world beliefs rather than general just-world beliefs fully mediated the effect of social support on goal pursuit, with a mediating effect of 39.00%. In conclusion, we found that for Chinese college students, social support has a significant positive effect on goal pursuit, and personal just-world beliefs play a fully mediating role in the effect of social support on goal pursuit.

## 1 Introduction

With the rise in popularity of motivational goal theory in the study of personality, the issue of goal pursuit has attracted widespread interest in psychology and has become a more active area of psychological research (Bernard et al., 2005; Diekman et al., 2017; Sheldon et al., 2018; Chen et al., 2019; Howard et al., 2021). College students are in the critical period of gaining maturity in their world views, life views, and values, and the pursuit of goals is reflected not only in their behavioral performance but also in their values. Therefore, examining the characteristics and influencing factors of their goal pursuit is very important for the successful exploration of self-identity and for promoting the good lifelong development of college students. Based on self-determination theory, this study investigated the mechanism of social support on college students’ goal pursuit, with special attention to the mediating role played by just-world beliefs.

Self-determination theory refers to the fact that a key issue affecting goal pursuit and achievement is the extent to which people are able to satisfy their basic psychological needs in the pursuit and attainment of valuable outcomes (Deci and Ryan, 1985; Gilal et al., 2019; Howard et al., 2021). Researchers have divided goals into two categories, i.e., intrinsic goals and extrinsic goals. Intrinsic goals involve the individual needs of self-development and growth, including self-acceptance, intimacy, group belonging, cooperation, public good, and physical health. The pursuit of intrinsic goals provides the intrinsic drive to meet these basic psychological needs as a way to promote personality integration, cognitive maturity, and self-actualization. Extrinsic goals, on the other hand, include matters such as obtaining extrinsic rewards or social approval and making a good impression on others, thus, making extrinsic values into goals to be pursued. The pursuit of extrinsic goals shows that an individual values money, reputation, prestige, and self-image, and the individual’s pursuit of these goals is more related to external standards. The pursuit of goals varies among individuals. Some researchers have found that compared to the pursuit of extrinsic goals, the pursuit of intrinsic goals is associated with higher happiness, better adjustment, and other positive outcomes. The pursuit of extrinsic goals is associated with lower happiness and poorer adjustment (Niemiec et al., 2009; Kasser, 2014).

Why do some people pursue extrinsic goals such as fame, money, and fortune, while others choose to pursue intrinsic goals such as intimacy and health? In a study of wellbeing, researchers found that support from intimate relationships facilitated individuals’ pursuit of goals that were conducive to self-growth (Feeney and Collins, 2014). That is, the availability of adequate support determined what goals individuals pursued. Researchers have also found that social support has a significant impact on goal pursuit. Social support is the feeling or experience that one is loved and cared for and respected and valued by others (Taylor, 2011). With the recent increased focus on mental health issues, there has been an increased interest in the impact of social support on family, society, peers, and adolescent development, particularly in the area of goal pursuit. Waaler et al. (2013) found that individuals in autonomously supported environments were more inclined to pursue intrinsic goals, while individuals with low levels of social support were more inclined to pursue extrinsic goals. Williams et al. (2000) also demonstrated that adolescents’ intrinsic life goal pursuits were positively correlated with their parents’ autonomy-supportive environment, i.e., the more autonomy support parents provided, the more likely children were to be inclined to establish intrinsic life goals, the less autonomy support parents provided, and the more likely children were to be inclined to establish extrinsic life goals. Lekes et al. (2010) also found that parental autonomy support facilitates the formation of children’s intrinsic goals in both Chinese and North American cultural contexts, suggesting cross-cultural consistency in this effect. Based on a previous study on the influence between social support and goal pursuit, the first hypothesis of this study is that there is a significant positive correlation between social support and goal pursuit.

Although numerous studies have found that social support can facilitate individuals’ intrinsic goal pursuit, the mechanisms are not clear. Just-world beliefs could play a role in the influence of social support on goal pursuit. Just-world beliefs refer to a view of justice that individuals hold about whether the real world is just or not, stemming from their intuitive judgments about justice and their social cognitive tendency to trust in justness (Lerner, 1980; Furnham, 2003; Bartholomaeus and Strelan, 2019). Depending on the object to which just-world beliefs are directed, they can be divided into general just-world beliefs and personal just-world beliefs. General just-world beliefs refer to individuals’ judgments about justice in their surroundings, i.e., the belief that the world is just for others; personal just-world beliefs are mainly concerned with justice in relation to oneself, i.e., the belief that the world is just for oneself.

The developmental socialization theory model suggests that the quality of adolescents’ social interpersonal relationships has a significant impact on the internalization of their beliefs and values (Grusec and Goodnow, 1994; Dalbert, 2001). Harmonious interpersonal relationships help to enhance cooperation, sharing, and trust between two individuals, allowing both individuals to feel justly treated (Gollwitzer and Prooijen, 2016). Therefore, social support could be a predictor variable of individuals’ just-world beliefs. Empirical studies have also found that social support is significantly related to just-world beliefs (Inman et al., 2015). DeConinck (2010) found a significant positive correlation between the perceived support of individuals in organizations and their just-world beliefs. The higher the level of social support is, the higher the level of organizational just-world beliefs perceived by individuals (Finkelstein et al., 2009). Dalbert and Radant (2004) also showed that individuals who live in an emotionally oriented and harmonious family atmosphere usually have higher levels of just-world beliefs. In addition to family support, peer support is also an important part of social support. Hafer and Bègue (2005) suggested that an individual’s belief in a just world is a “sense of entitlement” based on a “personal contract,” i.e., that the individual believes that he or she will be rewarded for what he or she gives and that others deserve to be rewarded for what they give. Social relationships between individuals depend on their interactions. The better the quality of friendships between individuals and their peers, the more interaction between the two sides in the process of communication. Individuals invest more time and energy in each other, which results in reciprocity from both sides as a result of their efforts. Therefore, they are more likely to have a stronger sense of “entitlement”; i.e., individuals are more likely to have a stronger sense that they are deserving of something. In particular, the individual can feel that he or she is being treated fairly. In summary, social support could have an impact on the formation of individuals’ beliefs about a just world.

Previous studies have shown that just-world beliefs are also related to individuals’ goal pursuits. Hafer (2000) found that just-world beliefs influence prosocial behavior, i.e., individuals with high levels of just-world beliefs are more likely to pursue intrinsic goals such as prosocial goals, suggesting that just-world beliefs can motivate individuals to focus on long-term goals. Additionally, Correia et al. (2016) found that volunteers’ motivation to help others can be positively predicted by personal just-world beliefs and self-efficacy to promote justice in the world. They also suggested that personal just-world beliefs are better predictor variables than general just-world beliefs. Based on the three-perspective model of interpersonal goal perception, helping behavior could be enabled by a cognitive approach that increases self-acquisition. Personal just-world beliefs reflect the individual’s perception that he or she is treated with justice and are also associated with a sense of self-acquisition. These results suggest that individuals with high levels of personal just-world beliefs could be more inclined to pursue intrinsic goals related to helping behavior.

**FIGURE 1:**
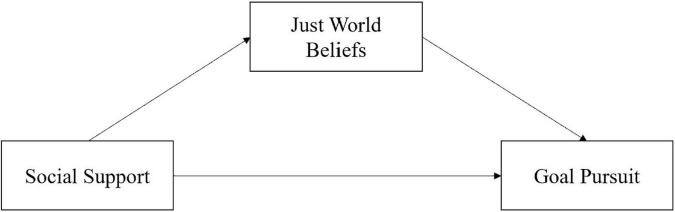
Theoretical model framework.

In summary, studies have shown that there is a relationship between social support, just-world beliefs, and goal pursuit; however, existing studies have not delved into the question of the mechanisms by which social support affects goal pursuit. This study is focused on Chinese college students because they are a group with rapidly developing and maturing concepts of autonomy whose goal pursuit has an important impact on the successful exploration of self-identity and good lifelong development. Young people will also become the backbone of society; therefore, the development of correct value orientation and the establishment of good goal pursuit during the critical period will form great social benefits, which can also greatly promote the positive development of society. Based on the above information regarding social support and just-world beliefs, the second hypothesis proposed in this study is that just-world beliefs play a mediating role between social support and goal pursuit (refer to Figure 1 for the theoretical model).

## 2 Materials and methods

### 2.1 Participants

In total, 424 college students were recruited by convenience sampling from several colleges and universities in Hangzhou, a large-size city located in southeastern China. Among them, 173 (40%) were male students, 251 (59%) were female students, 62 (14.6%) were freshmen, 95 (22%) were sophomores, 213 (50%) were juniors, and 54 (12%) were seniors. This study was approved by the Institutional Review Board at the local institution.

### 2.2 Measures

#### 2.2.1 Aspiration Index Scale

The Aspiration Index Scale developed by Kasser and Ryan (1996) was used in the current study. The Chinese version was revised by Tang et al. (2009). The scale contains two dimensions (intrinsic goals and extrinsic goals) and seven subscales. The intrinsic goals included four subscales of self-acceptance, group emotion, belongingness, and physical health; the extrinsic goals included three subscales of financial success, fame, and appearance attractiveness. Participants rated the importance of the content of the goals stated in each entry on a 7-point Likert scale, ranging from 1 (not at all important) to 7 (very important). To compare people’s value systems as a whole, the second-order factor scores for intrinsic values were subtracted from the second-order factor scores for extrinsic values to calculate the Relative Intrinsic vs. Extrinsic Value Orientation (RIEVO) scores (Kasser and Ryan, 1993). This approach was used because extrinsic values are not negative in themselves but are considered to be negative when they become more prominent than intrinsic values in an individual’s overall value system. Therefore, this study used the RIEVO score to describe the individual’s goal pursuit. Higher scores represent a greater pursuit of intrinsic goals by an individual. In this study, Cronbach’s alpha coefficient was 0.92.

#### 2.2.2 Just-World Beliefs Scale

The Just-World Beliefs Scale developed by Dalbert (1999) was used to measure the participants’ just-world beliefs. The Chinese version revised by Su et al. (2012) was used in the current study. The scale includes two dimensions, namely, general just-world beliefs and personal just-world beliefs, with a total of 13 items. Among them, there are 6 items that assess general just-world beliefs (GBJW) and 7 items that assess personal just-world beliefs (PBJW). All items were scored using a 6-point Likert scale, ranging from 0 (extreme disagreement) to 5 (extreme agreement). PBJW was obtained by the average scores of the corresponding 7 items, and GBJW was obtained by the average scores of the corresponding 6 items. The total score (BJW) was obtained by the average scores of PBJW and GBJW. The higher the score was, the higher the BJW obtained. In this study, Cronbach’s *a* coefficients were 0.94, 0.89, and 0.92 for the overall scale, the general just-world beliefs subscale, and the personal just-world beliefs subscale, respectively.

#### 2.2.3 Social Support Scale

The Perceived Social Support Scale (PSSS) was developed by Blumenthal et al. (1987). The Chinese version revised by Zhong et al. (2005) was used in the current study. The scale has 12 items and consists of three subscales, i.e., family support, friend support, and other supports (teachers, classmates, and relatives). This scale is a 7-point Likert scale with options ranging from 1 (extreme disagreement) to 7 (extreme agreement). The total social support score was obtained by adding up the scores of the three indicators; the higher the score, the higher the total social support obtained. In this study, Cronbach’s alpha coefficients for family support, friend support, other support, and the full scale were 0.86, 0.91, 0.87, and 0.93, respectively.

### 2.3 Procedure

An online questionnaire was generated using the Questionnaire Star platform,^1^ a widely used online questionnaire platform in China. Before filling out the questionnaires, the participants were briefed on the details and purpose of the study and their rights in the preface to the questionnaire. Participants then completed the basic demographic information, the Aspiration Index Scale, the Just-World Beliefs Scale, and the Social Support Scale. Each participant was given ¥5 as a reward after completing all questionnaires. SPSS 22.0 was used for statistical analysis. Independent samples *t*-tests and ANOVA were used to explore the relationships between demographic characteristics and study variables. Pearson correlations were utilized to investigate the correlations between variables, and stepwise regression was used to test the mediating effect.

## 3 Research results

### 3.1 Descriptive statistics and correlation analysis of social support, just-world beliefs, and goal pursuit

The means, standard deviations, and correlation matrices of the variables are shown in Table 1. Correlation analysis showed that there were significant positive correlations between social support, just-world beliefs, general just-world beliefs, personal just-world beliefs, and goal pursuit. We also compared the grade and gender differences in the three variables. Among different grades, we found a significant difference only in personal just-world belief [*F*(3,420) = 2.646, *p* < 0.05, $\eta_{p}^{2}$ 0.019]. *Post-hoc* analyses (LSD) found that juniors (*M* = 3.46, *SD* = 0.84) had significantly higher personal just-world beliefs than freshmen (*M* = 3.14, *SD* = 0.83, *p* < 0.01). Regarding the gender difference, independent sample *t*-test results showed that girls’ pursuit of external goals (*M* = 4.51, *SD* = 0.79) was significantly higher than boys’ (*M* = 4.29, *SD* = 0.91) [*t*(422) = 2.667, *p* < 0.01, Cohen’s *d* = 0.26]. We did not find any other grade or gender differences.

**TABLE 1:** Descriptive statistics.

	1	2	3	4	5	6	7	8	9	10	11	12
1. Gender(0 = Male; 1 = Female)	−	−	−	−	−	−	−	−	−	−	−	–
2. Grade	0.088	−	−	−	−	−	−	−	−	−	−	–
3. Social support	0.048	–0.035	−	−	−	−	−	−	−	−	−	–
4. Family support	0.020	–0.014	0.888***	−	−	−	−	−	−	−	−	–
5. Friend support	0.024	–0.015	0.875***	0.643***	−	−	−	−	−	−	−	–
6. Other support	0.084	–0.066	0.904***	0.711***	0.701***	−	−	−	−	−	−	–
7. Just-world beliefs	–0.013	0.064	0.504***	0.469***	0.442***	0.432***	−	−	−	−	−	–
8. Personal just-world beliefs	–0.018	0.076	0.519***	0.478***	0.460***	0.445***	0.930***	−	−	−	−	–
9. General just-world beliefs	–0.006	0.044	0.420***	0.396***	0.363***	0.359***	0.931***	0.733***	−	−	−	–
10. Extrinsic objectives	0.129**	–0.078	0.215***	−0.195***	0.113*	0.263***	0.093	0.082	0.091	−	−	–
11. Intrinsic objectives	0.063	–0.069	0.483***	0.389***	0.425***	0.477***	0.337***	0.329***	0.299***	0.416***	−	–
12. RIEVO	–0.081	0.024	0.169***	0.113*	0.226***	0.115*	0.176***	0.181***	0.147***	−0.684***	0.379***	–
*M*	0.59	2.61	5.09	4.95	5.34	4.99	3.30	3.37	3.23	4.41	5.81	1.39
SD	0.49	0.89	1.03	1.20	1.12	1.15	0.84	0.84	0.98	0.85	0.67	0.83

*p < 0.05, **p < 0.01, and ***p < 0.001.

### 3.3 Impact of social support on goal pursuit

The direct effect of social support on goal pursuit was examined first, and regression analysis revealed a significant positive effect of social support on the RIEVO score (β = 0.169, SE = 0.003, *t* = 3.518, *p* < 0.001). Therefore, Hypothesis 1 was confirmed.

### 3.4 Mediation model

**FIGURE 2:**
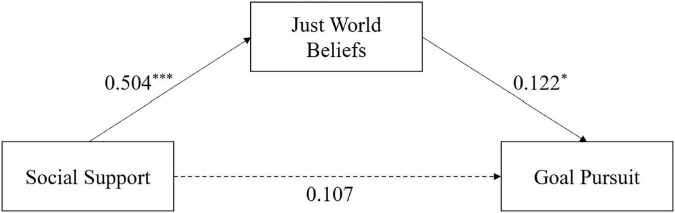
Mediating effects of just-world beliefs. *p < 0.05, **p < 0.01, and ***p < 0.001.

Then, we tested for the mediating effects of just-world beliefs on social support and goal pursuit (Table 2). The results showed that social support had a significant positive effect on just-world beliefs (β = 0.504, SE = 0.037, *t* = 11.987, *p* < 0.001) and that just-world beliefs had a significant positive effect on RIEVO (β = 0.122, SE = 0.004, *t* = 2.208, *p* < 0.05). While the effect of social support on RIEVO was not significant (*ß* = 0.107, SE = 0.004, *t* = 1.940, *p* = 0.053), just-world beliefs played a fully mediating role in the effect of social support on goal pursuit, with a mediating effect of 36.38% (Figure 2). Therefore, Hypothesis 2 was verified.

**TABLE 2:** Results of the regression for mediating effects of just-world beliefs.

Dependent variable	Independent variable	Regression test					
		
		${\text{R}}_{a\,d\,j}^{2}$	*F*	*B*	*β*	SE	*t*
RIEVO	Social support	0.026	12.377***	0.137	0.169	0.019	3.518***
Just-world beliefs	Social support	0.252	143.688***	0.414	0.504	0.035	11.987***
RIEVO	Social support	0.035	8.683***	0.087	0.107	0.045	1.940
	Just-world beliefs			0.121	0.122	0.055	2.208*

*p < 0.05, **p < 0.01, and ***p < 0.001.

**FIGURE 3:**
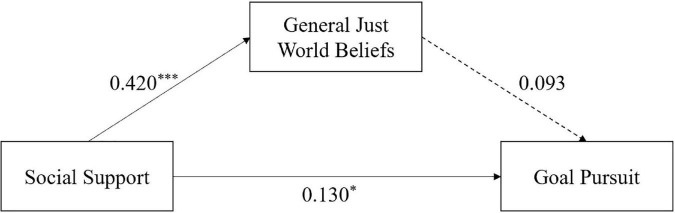
Mediating effects of general just-world beliefs. *p < 0.05, **p < 0.01, and ***p < 0.001.

To further test the potentially different roles of two dimensions of just-world beliefs, the two mediated models with general just-world belief and personal just-world belief as mediators were tested separately. The results showed that the mediating effect was not significant for general just-world beliefs (Table 3). Specifically, social support had a significant positive effect on general just-world beliefs (*ß* = 0.420, SE = 0.021, *t* = 9.497, *p* < 0.001). Social support also had a significant positive effect on RIEVO (*ß* = 0.130, SE = 0.004, *t* = 2.462, *p* < 0.05). General just-world beliefs had a nonsignificant effect on RIEVO (*ß* = 0.093, SE = 0.007, *t* = 1.762, *p* = 0.079) (Figure 3).

**TABLE 3:** Results of the regression for mediating effects of general just-world beliefs.

Dependent variable	Independent variable	Regression test					
		
		${\text{R}}_{a\,d\,j}^{2}$	*F*	*B*	*β*	SE	*t*
RIEVO	Social support	0.026	12.377***	0.137	0.169	0.039	3.518***
General just-world beliefs	Social support	0.174	90.200***	0.403	0.420	0.042	9.497***
RIEVO	Social support	0.031	7.772***	0.105	0.130	0.043	2.462*
	General just-world beliefs			0.079	0.093	0.045	1.762

*p < 0.05, **p < 0.01, and ***p < 0.001.

**FIGURE 4:**
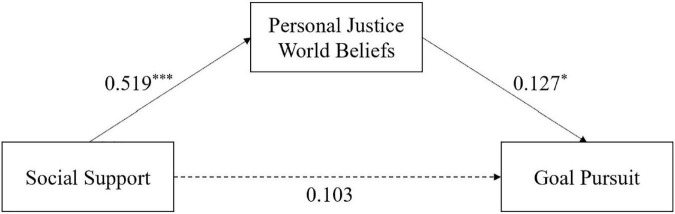
Mediating effects of personal just-world beliefs. *p < 0.05, **p < 0.01, and ***p < 0.001.

The mediating effect was significant for personal just-world beliefs (Table 4). Social support had a significant positive effect on personal just-world beliefs (*ß* = 0.519, SE = 0.020, *t* = 12.474, *p* < 0.001). Personal just-world beliefs had a significant positive effect on RIEVO (*ß* = 0.127, SE = 0.008, *t* = 2.281, *p* < 0.05). However, the effect of social support on RIEVO was not significant (*ß* = 0.103, SE = 0.004, *t* = 1.838, *p* = 0.067). Therefore, personal just-world beliefs fully mediated the effect of social support on goal pursuit (Figure 4). The mediating effect accounted for 39.00%.

**TABLE 4:** Results of the regression for mediating effects of personal just-world beliefs.

Dependent variable	Independent variable	Regression test					
		
		${\text{R}}_{a\,d\,j}^{2}$	*F*	*B*	*β*	SE	*t*
RIEVO	Social support	0.026	12.377***	0.137	0.169	0.039	3.518***
Personal just-world beliefs	Social support	0.268	155.613***	0.423	0.519	0.034	12.474***
RIEVO	Social support	0.036	8.852***	0.083	0.103	0.045	1.838
	Personal just-world beliefs			0.127	0.127	0.056	2.281*

*p < 0.05, **p < 0.01, ***p < 0.001.

## 4 Discussion

This study examined the relationship between college students’ social support and goal pursuit and examined the mediating role of college students’ just-world beliefs on social support and goal pursuit. The results showed that personal just-world beliefs, rather than general just-world beliefs, play a fully mediating role in the relationship between social support and goal pursuit.

### 4.1 The effect of social support on goal pursuit

The analysis of the relationship between social support and goal pursuit revealed that social support positively predicted goal pursuit, i.e., college students who had greater perceptions of social support were more likely to pursue intrinsic goals. This is consistent with Hypothesis 1 and is similar to the findings of Feeney and Collins (2014), who found that social support promotes individuals to pursue goals that are conducive to self-growth rather than extrinsic resources that can bring transient satisfaction. In contrast, Williams et al. (2000) and Lekes et al. (2010) also examined the relationship between parental autonomy support and children’s extrinsic goal pursuit from the early parent–child relationship perspective of social support and found that parents could encourage intrinsic life goals by being supportive of their children’s autonomy. In addition, Waaler et al. (2013) conducted a related study using high school students as participants and concluded that individuals in autonomy-supportive environments are more likely to pursue intrinsic goals. The previous studies are consistent with the results of the current study, possibly due to the cross-cultural consistency of the intrinsic and extrinsic goal structure dimensions–cross-cultural studies have shown that the goal structure dimension of intrinsic goals–extrinsic goals is cross-culturally consistent (Kim et al., 2003).

### 4.2 The mediating role of personal just-world beliefs

After clarifying that social support affects goal pursuit, we focused on the mechanism of the effect and found that just-world beliefs fully mediated the relationship between social support and goal pursuit, which was consistent with Hypothesis 2. Moreover, we also separately examined general just-world beliefs and personal just-world beliefs as mediators and found that personal just-world beliefs, rather than general just-world beliefs, played a fully mediating role. These results suggest that individuals with high levels of social support tend to believe that, for them, the world is just, which in turn facilitates individuals being more inclined to pursue their intrinsic goals. This finding reflects the mechanism of the relationship between social support and goal pursuit. Based on the developmental socialization theory model (Grusec and Goodnow, 1994; Dalbert, 2001), the quality of interpersonal relationships in adolescents can influence the formation of children’s value beliefs. Furthermore, we found that the values formed had an important influence on future goal pursuit. Adolescents who develop healthy and appropriate values are also more inclined to pursue intrinsic goals such as physical health and good intimate relationships, whereas adolescents who develop inappropriate values are more inclined to pursue extrinsic goals, such as money and fame, as they age (Williams et al., 2000; Lekes et al., 2010). The interpersonal relationships in children’s adolescence are peer relationships, parental relationships, and teacher-student relationships, all of which are important sources of social support for children. In addition, Gollwitzer and Prooijen (2016) showed that harmonious interpersonal social relationships help both interacting parties to increase their trust in each other and enable both individuals to be treated fairly, thus promoting the formation of just-world beliefs, which is also consistent with the results of this study. Meanwhile, there are discrepancies between the interpretations of the utility of PBJW and BJW results. Some studies have focused on the relationship between BJW and adaptive outcomes in China (Wu et al., 2011; Wu et al., 2013). Their conclusion is that in the context of collectivism, Chinese people pay more attention to group acquisition. This contradicts the conclusion of the current study. However, the current study focused on the relationship between BJW and goal pursuit, and the results showed that PBJW has better predictive power than BJW for goal pursuit. A plausible explanation for the contradiction might be that goal pursuit in the current study is more related to the individual. Although intrinsic goal pursuit is more likely to benefit the whole society in the Chinese context, the most direct utility is the improvement in individual ability and the realization of individual value. Therefore, the current study examined the structure of BJW from a new perspective.

### 4.3 Implications

In terms of theoretical significance, the current study is focused on Chinese college students, and it is the first study to introduce the mediating variable of personal just-world beliefs in the mechanism of how social support influences goal pursuit, which provides new empirical evidence and supports self-determination theory in different cultural backgrounds. Furthermore, the current study revealed that personal just-world beliefs, rather than general just-world beliefs, are more essential in influencing social support and goal pursuit, suggesting the need for subsequent research to focus on the individual’s concept of self. In terms of practical value, the mechanism of how social support affects goal pursuit could provide directional guidance for higher education reform in China. Previous results found that BJW could influence an individual’s adaptive functions (Donat et al., 2012) and subjective happiness (Donat et al., 2015), which suggested that BJW plays a significant role in education. When teaching college students which goals to pursue, educators should focus not only on giving them sufficient social support but should also demonstrate concern for students’ personal values, especially their personal just-world beliefs. At the same time, the conclusion of this study could be generalized to other settings, i.e., providing practical guidance for healthcare providers who are attempting to correct unreasonable behaviors and beliefs in their patients.

### 4.4 Limitations and direction for future research

One limitation of this study is that the data collected are all from college students’ self-reports. Since extrinsic goals such as the “pursuit of fame and fortune” are not widely accepted as positive social values in a particular culture, self-reported data could cause subjectivity and reliability issues. Future studies should try to collect data from multiple aspects, perspectives, and sources.

The other potential limitation is that the current study was cross-sectional. Although we found a significant correlation in the relationship between social support, just-world beliefs, and goal pursuit, the causal relationship between them should be investigated in depth. In the future, we could further explore the intrinsic causal relationship between the three variables using clinical intervention studies or longitudinal studies to obtain more robust findings.

## 5 Conclusion

This is the first cross-sectional study examining the mechanism of social support in influencing goal pursuit. The results showed that there is a significant positive correlation between social support, just-world beliefs, and goal pursuit in Chinese college students. We further tested the mediating role of just-world beliefs in the relationship between social support and goal pursuit and found that personal just-world beliefs, rather than general just-world beliefs, played a fully mediating role in the effect of social support on goal pursuit.

## Data availability statement

The raw data supporting the conclusions of this article will be made available by the authors, without undue reservation.

## Ethics statement

The studies involving human participants were reviewed and approved by Institute of Applied Psychology, Zhejiang University of Technology. The patients/participants provided their written informed consent to participate in this study.

## Author contributions

DC, YM, and YT designed the study. YM and YT collected the data. YM, YT, and XM analyzed the data. DC, XM, and YT wrote the manuscript. All authors contributed to the article and approved the submitted version.

## Footnotes

1
www.wjx.cn
